# A Standardized Extract of *Asparagus officinalis* Stem (ETAS^®^) Ameliorates Cognitive Impairment, Inhibits Amyloid β Deposition via BACE-1 and Normalizes Circadian Rhythm Signaling via MT1 and MT2

**DOI:** 10.3390/nu11071631

**Published:** 2019-07-17

**Authors:** Yin-Ching Chan, Ci-Sian Wu, Tsai-Chen Wu, Yu-Hsuan Lin, Sue-Joan Chang

**Affiliations:** 1 Department of Food and Nutrition, Providence University, Taichung 43301, Taiwan; 2 Department of Life Sciences, National Cheng Kung University, Tainan 70101, Taiwan

**Keywords:** ETAS^®^, cognitive impairment, circadian rhythm signaling, senescence-accelerated mice

## Abstract

The prevalence of cognitive impairments and circadian disturbances increases in the elderly and Alzheimer’s disease (AD) patients. This study investigated the effects of a standardized extract of *Asparagus officinalis* stem, ETAS^®^ on cognitive impairments and circadian rhythm status in senescence-accelerated mice prone 8 (SAMP8). ETAS^®^ consists of two major bioactive constituents: 5-hydroxymethyl-2-furfural (HMF), an abundant constituent, and (S)-asfural, a novel constituent, which is a derivative of HMF. Three-month-old SAMP8 male mice were divided into a control, 200 and 1000 mg/kg BW ETAS^®^ groups, while senescence-accelerated resistant mice (SAMR1) were used as the normal control. After 12-week feeding, ETAS^®^ significantly enhanced cognitive performance by an active avoidance test, inhibited the expressions of amyloid-beta precursor protein (APP) and BACE-1 and lowered the accumulation of amyloid β (Aβ) in the brain. ETAS^®^ also significantly increased neuron number in the suprachiasmatic nucleus (SCN) and normalized the expressions of the melatonin receptor 1 (MT1) and melatonin receptor 2 (MT2). In conclusion, ETAS^®^ enhances the cognitive ability, inhibits Aβ deposition and normalizes circadian rhythm signaling, suggesting it is beneficial for preventing cognitive impairments and circadian rhythm disturbances in aging.

## 1. Introduction

Cognitive impairment and circadian rhythm disturbances frequently occur in the elderly and is even more severe in Alzheimer’s disease (AD). AD is a progressive neurodegenerative disorder in the elderly, which is characterized by memory loss with multifactorial pathology. The brain of AD is marked by increased amyloid β (Aβ) deposits, hyperphosphorylated tau aggregates, synaptic losses, and inflammatory responses [1,2]. In vivo evidence indicates that Aβ impairs both long-term potentiation (LTP) and cognition [1]. Aggregated Aβ triggers the degeneration of septal neurons by phosphorylation of the tau protein induced neurodegeneration [3].

Amyloid-beta precursor protein (APP) is the precursor of Aβ, which is a functionally important molecule in its full-length configuration, as well as being the source of numerous fragments with varying effects on neural function [4]. In the normal condition, APP goes to the non-amyloidogenic pathway, which is cleaved by α-secretase and produces secreted APP (sAPP)α. When APP goes to the amyloidogenic pathway, it will form insoluble Aβ aggregation and produce plaques in the brain, which is the major pathological mark of AD [4,5,6]. Thus, decreased production and/or increased clearance of Aβ to reduce the Aβ deposition in the brain may be the promising strategy for preventing cognitive impairment. 

Amyloidosis causes not only cognitive impairment, but also leads to circadian rhythm disorders [7]. The prevalence of circadian rhythm disorders, such as sleep disturbances, is more pronounced in AD than in the elderly [8]. The suprachiasmatic nucleus (SCN), a small group of hypothalamic nerve cells in the brain, plays the role of a master circadian pacemaker controlling the timing of the sleep-wake cycle and coordinates with circadian rhythms in other brain areas and other tissues. Alterations in the SCN and the pineal gland during aging and AD are considered to be the biological basis for these circadian rhythm disturbances. Functional disruption of the SCN is observed from the earliest AD stages onwards. In addition, melatonin is noted to play an important role in regulating the circadian rhythms of both SCN and peripheral organs in mammals, which is largely mediated by the G-protein coupled membrane receptors melatonin receptor 1 (MT1) and MT2 [9,10,11]. In late clinical AD patients, the number of MT1 receptor containing neurons in the SCN is only 10% of those in age-matched control subjects [8]. 

*Asparagus* (*Asparagus officinalis* L.) has been proven to have many physiological benefits, such as antioxidant [12], anti-tumor [13], and lower blood levels of lipid and glucose [14,15]. ETAS^®^, a standardized extract of *Asparagus officinalis* stem, consists of two major bioactive constituents: 5-hydroxymethyl-2-furfural (HMF), an abundant constituent, and (S)-asfural, a novel constituent, which is a derivative of HMF. Sakurai et al. [16] demonstrated that treated NG108-15 neuronal cells with ETAS^®^, enhanced the heat shock protein 70 (HSP70) and HO-1 mRNA expressions by increasing the transcriptional activities of heat shock factor 1 (HSF1) and Nrf2, and lessened the cell damage. They further examined the memory performance of senescence-accelerated prone (SAMP8) mice, and found that ETAS^®^ significantly increased the ratio of freezing responses, whereas the cued fear memory was not affected. However, the underlying mechanisms of ETAS^®^ on active avoidance response and circadian rhythm signaling has not been investigated yet. Thus, this study aimed to evaluate the effects of ETAS^®^ on cognitive status by an active avoidance test, and investigated the signaling of Aβ and circadian rhythm in aging using SAMP8 mice.

## 2. Materials and Methods

### 2.1. Animals and Diet

SAMP8 (SAMP8/Ta Slc) and senescence-accelerated-resistant (SAMR1/Ta Slc) mice were acquired from the Council for Senescence-Accelerated Mouse (SAM) Research, Japan, and were maintained through inbreeding in the standard animal room at Providence University. ETAS^®^, a trademark of Amino Up Co., Ltd., Sapporo, Japan, was kindly supplied by the company. Three-month-old male SAMP8 mice were randomly divided into a control (SAMP8 control group) and two ETAS^®^ groups, while SAMR1 mice (SAMR1 group) were used as the external control (n = 8/group). The two control groups were fed an American Institute of Nutrition (AIN) 93-M basal diet, while the ETAS^®^ groups were fed an AIN 93-M basal diet supplemented with 200 and 1000 mg/kg BW/day of ETAS^®^, respectively. The mice were housed under controlled environmental conditions (22 ± 2 °C, 65% ± 5% relative humidity, 7:00–19:00 lighting period). Animals were allowed free access to drinking water and experimental diets for 12 weeks. The study protocol was approved by the Animal Research Ethics Committee at Providence University, Taichung, Taiwan (20180605-P002).

### 2.2. Cognitive Evaluation

Cognitive performance was evaluated by active avoidance testing as described in our previous study [17]. In brief, the successful avoidance response was evident if the tested mouse moved itself from one compartment to the other compartment within the shuttle box after receiving a conditional stimulus (CS) consisting of 10 s of tone and red, yellow, and green light, and the avoidance responses were automatically recorded. The test was conducted for 2 days, and each mouse received four daily sessions comprised of 5 trials for a total of 20 trials. 

### 2.3. Measurements of Amyloid β (Aβ)

Aβ was measured as described in our previous study [17]. Brain tissue was quickly dissected after being sacrificed, fixed in a 10% buffered neutral formalin solution for one week, and then sectioned. Brain sections were incubated with primary anti-β amyloid antibody (1:100; anti-β-amyloid 1–42 antibody, Merck millipore, Darmstadt, Germany) for 2 h, incubated in polyvalent-biotinylated antibody (goat anti-rabbit antibodies, 1:1000) for 45 min, incubated in 3,3′Diaminobenzidine (DAB) substrate solution for 30 min, and stained with Mayer’s hematoxylin (Sigma, St. Louis, MO, USA) for 3 min. The Aβ positive areas, appeared brownish in color, in sections of the hippocampus and whole brain and were measured under 100× magnification by an image analyzer (Leica, Q500MC, Nussloch, Germany).

### 2.4. Morphometric Analysis of the Suprachiasmatic Nucleus (SCN)

SCN was evaluated according to the description of Engelberth et al. [18]. In brief, six images (objective 40×) of each animal, one at the rostral level, three at the medium level, and two at the caudal level, representative of the rostrocaudal extension of area of interest, were selected to count the number of SCN cells. Histological characteristics presented by neurons in the thionine dye, included rounded shape, strong staining, and evident nucleolus and were used as selection criteria in the cell counting.

### 2.5. Western Blotting 

Western blotting was performed as described in our previous study [19]. Total protein samples were extracted from brain tissues, and their protein concentrations were evaluated by the bicinchoninic acid (BCA) method (Bio-Rad, Hercules, CA, USA). Protein electrophoresed on 10% (*w*/*v*) SDS polyacrylamide gels were transferred onto polyvinylidene difluoride (PVDF) membranes, and blocked with TBST (TBS with 0.1% Tween-20) containing a 5% nonfat dry milk or 5% bovine serum albumin (BSA) for 1 h. After blocking, membranes were kept overnight at 4 °C in blocking solution containing different primary antibody, including anti-APP (1:6000, Abcam, Cambridge, MA, USA), anti-BACE-1 (1:10,000, Abcam, Cambridge, MA, USA), anti-MT1 (1:1000, IBL, Hamburg, Germany), anti-MT2 (1:6000, Abcam, Cambridge, MA, USA), and anti-β actin (1:50,000, CSL, USA) antibodies. The membranes were exposed to secondary antibodies after being washed with TBST at room temperature for 1 h. Immunoreactivity was detected using enhanced chemiluminescence (ECL) reagents. Quantitative results were performed using a FusionCapt Advance Camera and FusionCapt Advance Analyzer (version 16.07, Sursee, Switzerland).

### 2.6. Statistics

Data were expressed as mean ± standard error of mean (SEM) and were analyzed using SPSS software (SPSS Inc., Chicago, IL, USA). Differences were evaluated using one-way analysis of variance. Student’s t-test was used for comparisons between groups when the F-test was significant. Differences were considered to be significant at the *p* < 0.05 level.

## 3. Results 

### 3.1. ETAS^®^ Improved Cognitive Function

**Figure 1:**
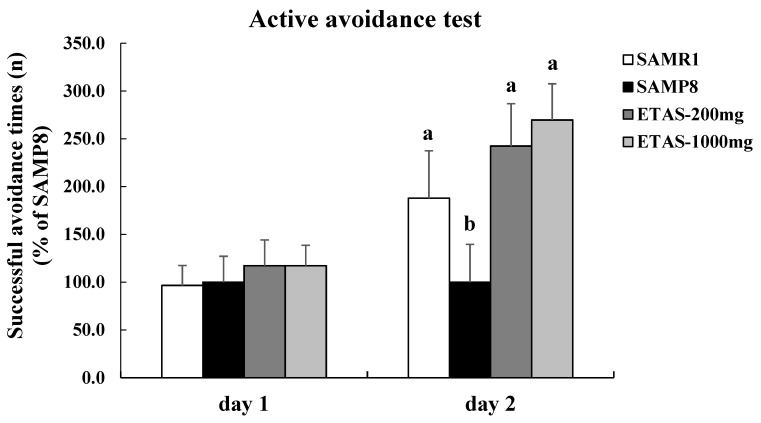
Mean number of successful active avoidance among 20 trials of shuttle avoidance test in 3-month-old SAMP8 mice fed with a different diet for 12 weeks. The y axis denotes the percentage of each group compared to SAMP8 control group. Values were mean ± S.E.M. (n = 8/group). Data with different superscripts are significantly different among groups.

ETAS^®^ significantly increased the successful active avoidance times on the next day in SAMP8 mice (Figure 1). Moreover, there was no significant difference in the successful active avoidance times between the SAMP8 mice fed ETAS^®^ groups and the SAMR1 control group (*p* > 0.05). These results indicated that supplementation of ETAS^®^ to the diet improved the cognitive impairment in SAMP8 mice.

### 3.2. ETAS^®^ Reduced the Expressions of APP, BACE-1 and Aβ Deposition

**Figure 2:**
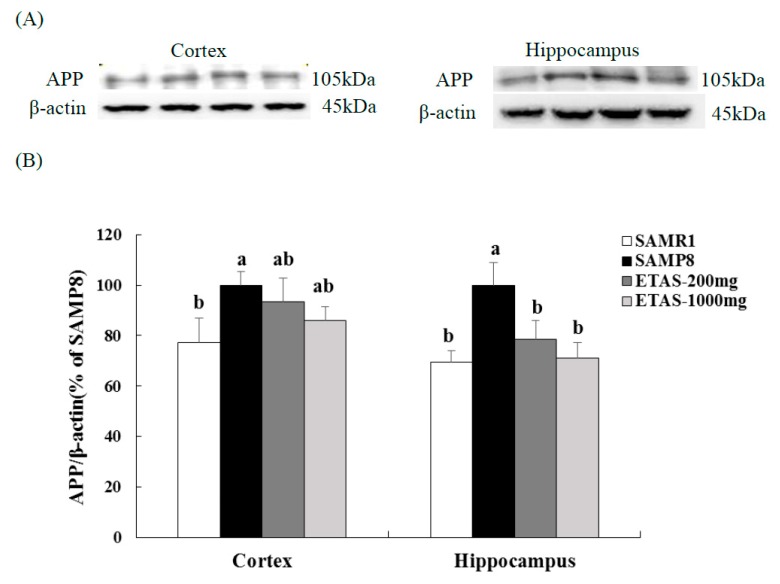
APP expressions in the cortex and hippocampus of 3-month-old male mice fed with a different diet for 12 weeks. (**A**) Electrophoretograms of APP and β-actin expressions. (**B**) APP expressions of different groups. The y axis denotes the percentage of each group compared to SAMP8 control group. Values were mean ± S.E.M. (n = 8/group). Data with different superscripts are significantly different among groups.

**Figure 3:**
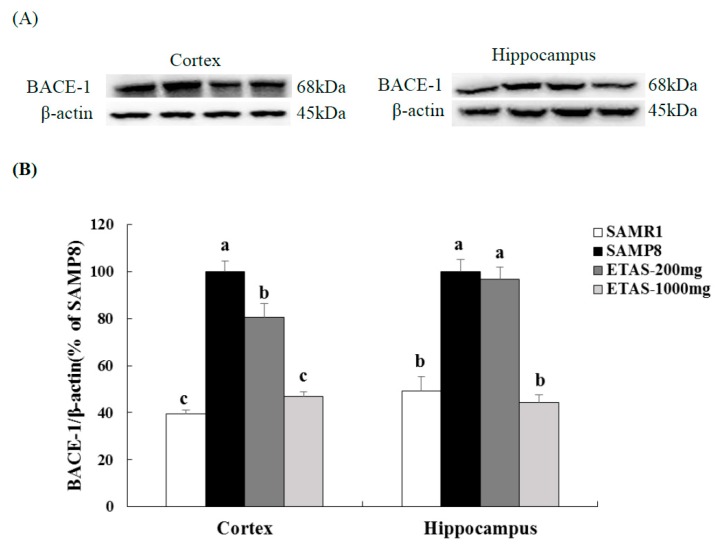
BACE-1 expressions in the cortex and hippocampus of 3-month-old male mice fed with a different diet for 12 weeks. (**A**) Electrophoretograms of BACE-1 and β-actin expressions. (**B**) BACE-1 expressions of different groups. The y axis denotes the percentage of each group compared to SAMP8 control group. Values were mean ± S.E.M. (n = 8/group). Data with different superscripts are significantly different among groups.

**Figure 4:**
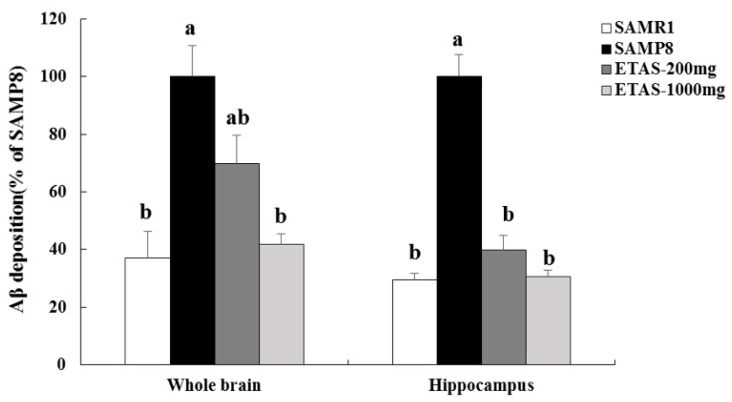
Aβ depositions in the whole brain and hippocampus of 3-month-old SAMP8 mice fed different diets for 12 weeks. Aβ accumulation was assessed under 100× magnification. The y axis denotes the percentage of each group compared to the SAMP8 control group. Values were mean ± S.E.M. (n = 8/group) Data with different superscripts are significantly different among groups.

SAMP8 mice fed ETAS^®^ had lower APP and BACE-1 levels in the cortex and hippocampus when compared with the SAMP8 control group (Figure 2 and Figure 3). The 1000 mg/kg BW ETAS^®^ supplementation significantly decreased levels of APP and BACE-1 to that in the SAMR1 normal control group. Furthermore, depositions of Aβ in the hippocampus and whole brain were lower in both ETAS^®^ groups than that in the SAMP8 control group, while a strong significance was found in the 1000 mg/kg BW ETAS^®^ group (Figure 4). These results indicated that ETAS^®^ decreased Aβ accumulation by downregulating the APP and BACE-1 expressions.

### 3.3. ETAS^®^ Increased SCN Neuron and Improved the Circadian Rhythm Modulators

**Figure 5:**
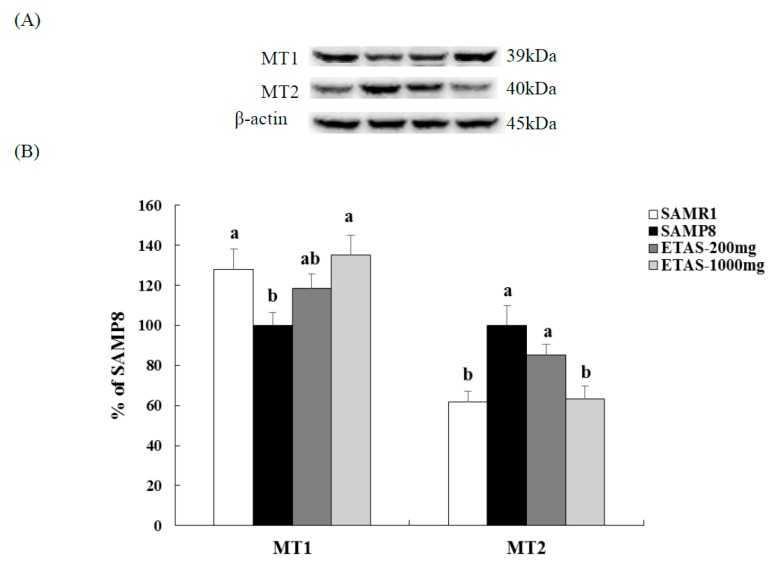
MT1 and MT2 expressions in the hypothalamus of 3-month-old male mice fed with a different diet for 12 weeks. (**A**) Electrophoretograms of MT1, MT2 and β-actin expressions. (**B**) MT1 and MT2 expression of different groups. The y axis denotes the percentage of each group compared to the SAMP8 control group. Values were mean ± S.E.M. (n = 8/group). Data with different superscripts are significantly different among groups.

**Figure 6:**
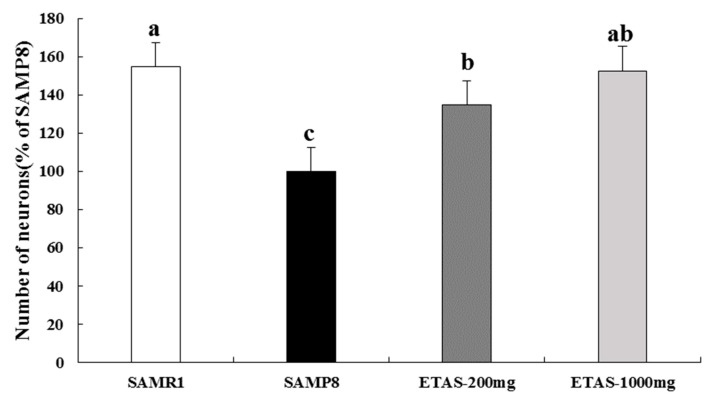
Neuron numbers in the suprachiasmatic nuclei of 3-month-old male mice fed with a different diet for 12 weeks. Neuron numbers of Nissl staining was assessed under 10× magnification. The y axis denotes the percentage of each group compared to the SAMP8 control group. Values were mean ± S.E.M. (n = 8/group). Data with different superscripts are significantly different among groups.

SAMP8 mice supplemented with 1000 mg/kg BW ETAS^®^ had significantly higher MT1 and lower MT2 levels than the SAMP8 control group, which were comparable with those in the SAMR1 group, while the 200 mg/kg BW ETAS^®^ group had a similar tendency (Figure 5). Moreover, neuron numbers of SCN in both two ETAS^®^ groups were significantly higher than that in the SAMP8 control group, and similar to the SAMR1 group (Figure 6). These data suggested that ETAS^®^ reduced the neuron loss of the master circadian pacemaker—SCN and normalized the circadian rhythm mediators—MT1 and MT2. 

## 4. Discussion

This study demonstrated that ETAS^®^ improved cognitive performance, and lowered Aβ deposition by reducing APP and BACE-1 levels in SAMP8 mice. ETAS^®^ also increased neuron number in the SCN, and normalized the MT1 and MT2 expressions. These results indicate ETAS^®^ as a beneficial agent for improving the cognitive impairments and circadian rhythm disturbances in aging.

SAMP and SAMR are two accelerated senescence lines of in inbred mice developed for studying the aging process [20]. Within strains of SAMP, SAMP8 mice exhibit an age-related decline in learning and memory abilities (passive and active avoidance), while the SAMR1 does not reveal significant changes [21,22,23,24]. In addition, SAMP8, characterized by an impaired circadian timing [25], has similar age-related changes in the circadian rhythms to those reported in aged humans and patients with senile dementia [26,27]. These data support the use of the SAMP8 as a model for cognitive deficits and circadian rhythm disruptions associated with senile dementia.

Circadian rhythm disorders, such as sleep disturbances, are associated with aging and even more pronounced in AD, and these changes may be due to the degeneration of the retina-SCN-pineal axis [28]. Patients diagnosed with an SCN lesion showed a disturbed circadian rhythm [29], while the AD patients had more extensive cell loss in the SCN [30]. Under the control of the SCN, melatonin is secreted from the pineal gland, and further delivers important feedback to the SCN. The dysfunction of the melatonin system is associated with the stability of the circadian rhythm, and the membrane receptor melatonin receptor (MT) is thought to be involved in the feedback of melatonin on the SCN rhythm. The MT1 receptor was noted to affect the excitability of SCN neurons as the clock transferred from day into night, while the MT2 melatonin receptor affected the clock phase shift at dusk (day–night) and dawn (night–day). The expression of MT1 in SCN decreases with age, especially in AD [8,31,32]. Through normalizing the MT2, melatonin mediates phase advances in the SCN circadian clock of rats [33], and diminishes the isoflurane-induced cognitive impairment in aged rats [34]. The aforementioned reveals the importance of SCN, MT1, and MT2 on the normalization of the circadian rhythm. MT1 and MT2 mRNAs are confirmed to be expressed in the SCN of both SAMR1 and SAMP8 strains by RT-PCR techniques [35]. MT1 protein level reduces with age in the whole brain of SAMR1 and SAMP8 with marked and earlier reduction in SAMP8 by western blotting detection [36]. To our knowledge, the MT2 protein levels in the SCN of SAMR1 and SAMP8 have not been detected yet. In this study, we were the first to investigate the MT2 protein level in the SCN of SAMR1 and SAMP8. Activation of MT2 mediated phase advances of the SCN circadian clock at both dusk and dawn [33], which may contribute to the disturbance of the sleep-wake cycle. Our results demonstrated that SAMP8 had lower MT1 and higher MT2 protein levels in SCN than the SAMR1 group. ETAS^®^ increased the MT1 and reduced the MT2 levels, and reduced the cell loss in the SCN, especially 1000 mg/kg BW ETAS^®^ recovered MT1, MT2 and cell numbers to those in the normal control SAMR1. These results indicate that ETAS^®^ is beneficial for the disturbed circadian rhythm in aging.

Aβ accumulation has been widely recognized as one of the main characteristics of AD. Aβ is produced from the sequential cleavage of APP by β-secretase and γ-secretase [37]. BACE-1 is the main β-secretase that is involved in the Aβ-generating process and plays a critical role in Aβ production and amyloidosis in the central nervous system [38,39]. BACE-1 cleaves the ectodomain of APP and generates an APP C-terminal fragment. Then, γ-secretase cleaves the transmembrane domain and releases Aβ [40]. Thus, APP and BACE-1 are highly associated with Aβ production. 

SAMP8 mice are described with age-related increases in Aβ and APP [41]. Several studies have investigated the effects of different bioactive components on the Aβ, APP and BACE-1 levels in SAMP8 mice. Docosahexaenoic acid (DHA)-enriched phosphatidylcholine and eicosapentaenoic acid (EPA)-enriched phosphatidylcholine lowers the cognitive deficit and decreases the Aβ level of SAMP8 mice by inhibiting APP and BACE-1 levels [42]. Panax notoginseng saponins reduces the APP and BACE-1 levels, and inhibits the Aβ generation in the brain of SAMP8 mice [43]. Pseudoginsenoside-F11 lessens the impairments of learning and memory in SAMP8 mice, promotes the APP transporting from the cytoplasm to plasma membrane and diminishes the BACE-1 expression in the hippocampus and cortex [44]. In the present study, our results indicated that ETAS^®^ lowered APP and BACE-1 expressions and reduced Aβ accumulation in the brain of SAMP8 mice. 

*Asparagus* (*Asparagus officinalis* L.) contains abundant bioactive components, such as flavonoids and carotenoids [45,46]. Flavonoids may reduce the Aβ gathering and then achieve a neuroprotective effect [47]. Hesperetin, a predominant flavonoid in lemons and oranges, improves learning and memory impairment by enhancing the antioxidant defense and BDNF signaling [47]. The neuroprotective effects of quercetin are associated with regulating the nuclear factor (erythroid-derived 2)-like 2 (Nrf2), mitogen-activated protein kinase (MAPK) signalling cascades, and PI3K/Akt pathways [48]. Neural stem cells pretreated with lycopene enhanced BDNF level, reduces the oxidative stress and t-BHP-induced cell apoptosis [49]. Three carotenoids, cryptocapsin, cryptocapsin-5,6-epoxide, and zeaxanthin, show anti-amyloidogenic activity through preventing the fibril formation via disruption of the Aβ aggregates [50]. This evidence supports the potential for foods rich in bioactive ingredients to prevent or reverse age-dependent deterioration.

ETAS^®^, a standardized extract of *Asparagus officinalis* stem, is noted to reduce the lipid peroxides in sleep deprived/stress-loaded mice [51]. ETAS^®^ administration improves the balance of the autonomic nerve, decreases psychological stress and early-morning awakening in healthy subjects with mild sleep anxiety [52], and helps to stabilize emotional stress and sleep quality [53]. ETAS^®^ reduces the injury of the NG108-15 neuronal cell by increasing the levels of HSP70 and HO-1 mRNA and enhances memory performance by a freezing response test in SAMP8 mice [16]. ETAS^®^ has been reported to suppress the levels of pro-inflammatory cytokines and chemokines in hepatocytes of rats [54], and to prevent pro-inflammatory responses in H202-induced skin fibroblasts [55], which may alleviate neuroinflammation involved in the aging progress and the initial stage of AD [56,57]. The study regarding the efficacy of ETAS^®^ on the expression of anti-inflammation related lymphokines leading to the improvement of cognition was warranted and limited in this study due to the constraints on brain samples. In the present study, ETAS^®^ improved cognitive status via APP/BACE/Aβ, and normalized the signaling of the circadian rhythm via MT1/MT2. These findings suggest that ETAS^®^ is beneficial for cognitive impairments and circadian disturbances.

## 5. Conclusions

We were the first to indicate the MT2 protein level in the SCN of SAMR1 and SAMP8. Our results revealed that ETAS^®^ enhanced the successful active avoidance performance, reduced Aβ generation/deposition and normalized circadian rhythm mediators, MT1 and MT2. This evidence supports the potential for ETAS^®^ to be used to lessen the pathological progresses of cognitive impairment, decrease Aβ deposition, and improve circadian rhythm signaling in aging.
